# Rhizosphere ecology of lumichrome and riboflavin, two bacterial signal molecules eliciting developmental changes in plants

**DOI:** 10.3389/fpls.2015.00700

**Published:** 2015-09-14

**Authors:** Felix D. Dakora, Viviene N. Matiru, Alfred S. Kanu

**Affiliations:** ^1^Department of Chemistry, Tshwane University of Technology, Pretoria, South Africa; ^2^Department of Botany, Jomo Kenyatta University of Agriculture and Technology, Nairobi, Kenya; ^3^Department of Agriculture and Animal Health, School of Agriculture and Environmental Sciences, University of South Africa, Florida, South Africa

**Keywords:** plant growth promoting molecules, IAA, ABA, rhizosphere, rhizobial exudates

## Abstract

Lumichrome and riboflavin are novel molecules from rhizobial exudates that stimulate plant growth. Reported studies have revealed major developmental changes elicited by lumichrome at very low nanomolar concentrations (5 nM) in plants, which include early initiation of trifoliate leaves, expansion of unifoliate and trifoliate leaves, increased stem elongation and leaf area, and consequently greater biomass accumulation in monocots and dicots. But higher lumichrome concentration (50 nM) depressed root development and reduced growth of unifoliate and second trifoliate leaves. While the mechanisms remain unknown, it is possible that lumichrome released by rhizobia induced the biosynthesis of classical phytohormones that caused the observed developmental changes in plants. We also showed in earlier studies that applying either 10 nM lumichrome, 10 nM ABA, or 10 ml of infective rhizobial cells (0.2 OD_600_) to roots of monocots and dicots for 44 h produced identical effects, which included decreased stomatal conductance and leaf transpiration in Bambara groundnut, soybean, and maize, increased stomatal conductance and transpiration in cowpea and lupin, and elevated root respiration in maize (19% by rhizobia and 20% by lumichrome). Greater extracellular exudation of lumichrome, riboflavin and indole acetic acid by N_2_-fixing rhizobia over non-fixing bacteria is perceived to be an indication of their role as symbiotic signals. This is evidenced by the increased concentration of lumichrome and riboflavin in the xylem sap of cowpea and soybean plants inoculated with infective rhizobia. In fact, greater xylem concentration of lumichrome in soybean and its correspondingly increased accumulation in leaves was found to result in dramatic developmental changes than in cowpea. Furthermore, lumichrome and riboflavin secreted by soil rhizobia are also known to function as (i) ecological cues for sensing environmental stress, (ii) growth factors for microbes, plants, and humans, (iii) signals for stomatal functioning in land plants, and (iv) protectants/elicitors of plant defense. The fact that exogenous application of ABA to plant roots caused the same effect as lumichrome on leaf stomatal functioning suggests molecular cross-talk in plant response to environmental stimuli.

## Introduction

In nature, plants and soil microbes seem to have co-evolved to overcome environmental stress in their habitats. Outside pathogenesis and allelopathy, many plant–plant or plant–bacterial interactions have tended to be facilitative in providing benefits to both partners (He et al., 2013). Thus, the rhizosphere is generally regarded as the hotspot of interactive events between soil microbes and plants, which occur through perception of signals released in the form of simple chemical molecules. In nutrient-poor soils, a typical rhizosphere consists of mixtures of molecules secreted by both plants and microbes for promoting nutrient mobilization and increased mineral uptake (Marschner, 1995; Dakora and Phillips, 2002). Under Fe-limiting conditions, bacterial species can secrete specialized compounds such as siderophores to enhance Fe acquisition (Jurkevitch et al., 1986). In times of abiotic stress such as drought, soil microbes, including rhizobia and other diazotrophs, produce chemical molecules in their exudates that effect changes in plant development. In an exhaustive review, Mehboob et al. (2009) found that applying 29 different rhizobial species/strains to 11 non-legume crops led to an increase in plant growth, plant height and plant biomass, as well as greater tissue concentration of N, P, K, Ca, Mg, Na, Fe, Zn, and Cu in plant organs. The growth-promoting molecules released by the test rhizobia included indole acetic acid (IAA) by 13 strains, gibberellins by four strains, exopolysaccharides by three strains, followed by lipopolysaccharides, hydrocyanic acid, abscisic acid (ABA), phenolics and lumichrome by one strain each. This review assesses lumichrome and riboflavin, and to some extent IAA, as rhizobial signals influencing plant growth, and discusses their roles in the rhizosphere of monocots (cereals) and dicots (legumes) in relation to plant growth and mineral nutrition.

## Molecular Signals From Rhizobial Exudates and Their Effects on Plant Growth

**FIGURE 1:**
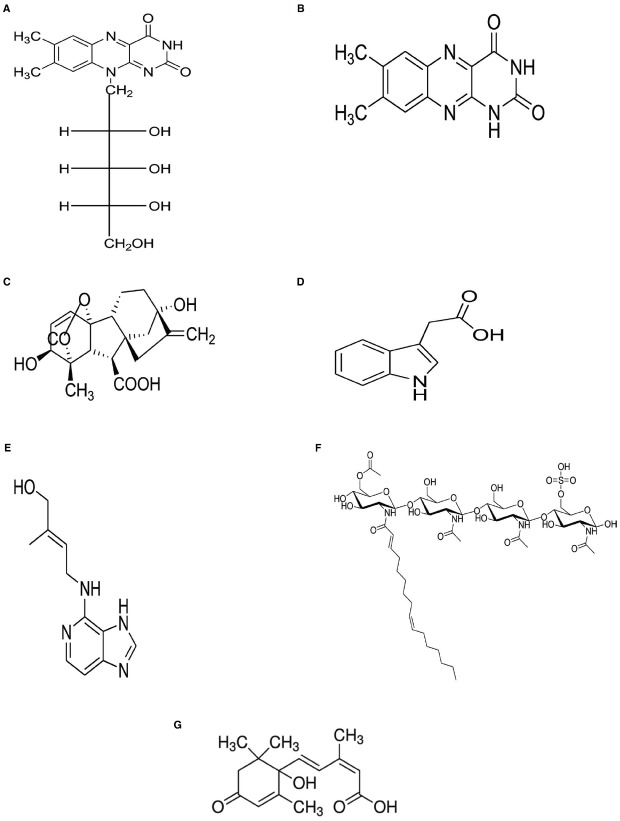
**Structures of selected rhizobial molecules functioning as plant growth promoters. (A)** Riboflavin, **(B)** Lumichrome, **(C)** Indole acetic acid, **(D)** Gibberelline, **(E)** Cytokinins, **(F)** Nod factors, and **(G)** Abscisic acid.

Species and strains of rhizobia are reported to synthesize various metabolites for bacterial cell growth. These include the vitamins thiamine, niacin, biotin, ascorbic acid, and pantothenic acid, as well as the amino acids glutamate, lysine, arginine, tryptophan, and methionine purified from culture filtrates of *Sinorhizobium meliloti*, *Rhizobium leguminosarum* bv. *viceae*, *Azospirillum brasilense*, *Azotobacter vinelandii*, and *Pseudomonas fluorescens* (Rodelas et al., 1993; Sierra et al., 1999; Yang et al., 2002). In addition to IAA, simple nitrogenous molecules such as cytokinins, gibberellins, lumichrome, and riboflavin (Figure 1) have also been purified from bacterial culture filtrates and proven to be active signals controlling plant development. Gibberellins and cytokinins isolated from symbiotic rhizobia (Phillips and Torrey, 1970, 1972; Dart, 1974; Lynch and Clark, 1984) are known to promote bacterial cell growth, as well as stimulate root hair production in plants for increased uptake of water and mineral nutrients (Yanni et al., 2001).

In addition to traditional bacterial hormones such as gibberellins, cytokinins and IAA, lipo-chitooligosaccharide molecules (Figure 1) represent a new group of biologically-active compounds that stimulate cell growth and induce nodule organogenesis (De Jong et al., 1993; Dyachok et al., 2000). In the absence of rhizobial cells, purified Nod factors can morphogenically elicit nodule formation in legumes (Dénarié et al., 1996). Furthermore, exogenously applied rhizobial Nod factors have been reported to stimulate seed germination (Zhang and Smith, 2002) and promote seedling development in both monocots and dicots (Smith et al., 2002). Applying Nod factors (10^–7^ M or 10^–9^ M) to soybean plants increased root mass by 7–16%, and root length by 34–44% (Smith et al., 2002). Similarly, spraying sub-micromolar concentrations (10^–6^, 10^–8^, or 10^–10^ M) of Nod factors on leaves of soybean, common bean, maize, rice, canola, apple, and grape plants increased photosynthetic rates by 10–20%, and caused a 40% increase in grain yield of field-grown soybean (Smith et al., 2002). But more importantly, Nod factors also induce the expression of genes involved in the phenylpropanoid pathway (Savouré et al., 1994; Spaink and Lugtenberg, 1994), and in so doing increase phytoalexin biosynthesis, which has the potential to protect the host plant against pathogens (Dakora and Phillips, 1996). It has also been shown that, even at low concentrations (10^–7^ nM), Nod factors can promote AM colonization of both nodulating and non-nodulating plants (Xie et al., 1995), suggesting a role for this rhizobial metabolite in the establishment of mycorrhizal symbiosis (Parniske, 2008). In fact, it has now been shown that after the initial Nod factor and Myc factor perception, both nodulation and mychorrhization processes share a common symbiotic pathway (Parniske, 2008; Maillet et al., 2011).

Although lumichrome is considered a novel molecule that stimulates plant development (Phillips et al., 1999; Matiru and Dakora, 2005a), the discovery that rhizobia are capable of synthesizing riboflavin (a precursor of lumichrome biosynthesis) and thiamine for cell growth occurred almost 80 years ago (West and Wilson, 1938). Carpenter (1943) later isolated riboflavin from field soil and showed its uptake by plant roots and translocation to shoots. However, the role of this molecule in plant growth stimulation was only reported thirty five years later (Rao, 1973). Today, the findings of new studies have shown that lumichrome and riboflavin purified from rhizobial exudates can promote plant growth and alter stomatal function (Phillips et al., 1999; Matiru and Dakora, 2005a,b). What was however unclear is whether the exudation of lumichrome and riboflavin is unique to N_2_-fixing rhizobia.

## Lumichrome and Riboflavin are Symbiotic Signals Involved in Plant Development

**FIGURE 2:**
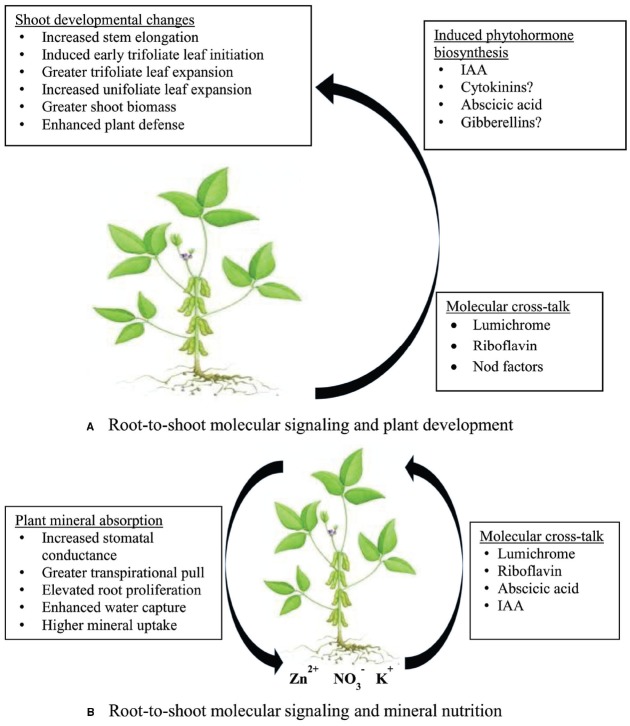
**A model describing the effect of root-to-shoot signaling by rhizobial molecules on (A) shoot developmental changes, and (B) symbiosis-induced mineral uptake in the rhizosphere**.

Lumichrome is a molecule commonly synthesized by both microbes and plants, but is also a known degradation product of the vitamin riboflavin (Phillips et al., 1999). As a result, the role of lumichrome is often linked with riboflavin as the latter is easily converted enzymatically or photochemically into lumichrome (Yanagita and Foster, 1956; Yagi, 1962). Applying purified lumichrome from *S. meliloti* exudates to roots of alfalfa seedlings increased root respiration by 11–30%, and promoted plant growth by 8–18% (Phillips et al., 1999). The enhanced plant growth was attributed to increased net C assimilation, possibly via PEP carboxylase activity (Phillips et al., 1999). Later studies have shown that plants exhibit a mixed response to lumichrome and riboflavin application (see Figure 2A). While this molecule significantly increased root respiration in maize plants (Phillips et al., 1999; Matiru and Dakora, 2005b), it decreased it in lupin, and had no affect on cowpea, soybean, Bambara groundnut, pea, and sorghum (Matiru and Dakora, 2005b). Inoculating the roots of these monocots and dicots with ineffective rhizobial cells produced the same results as obtained with lumichrome application, in that, maize showed significantly increased rate of root respiration, and lupin a decreased rate, while cowpea, soybean, Bambara groundnut, pea, and sorghum were unaffected in their root respiration (Matiru and Dakora, 2005b). These responses by both monocots and dicots to rhizobia and purified lumichrome clearly indicate that the observed changes in root respiration with bacterial inoculation were caused by lumichrome released by the applied rhizobia. Other studies have similarly found increased root respiration and dry matter accumulation following lumichrome supply to lotus and tomato (Gouws et al., 2012). Furthermore, both lumichrome and riboflavin have been implicated as quorum-sensing molecules in rhizobial bacteria (Rajamani et al., 2008). But the independent role of riboflavin as a signal molecule was underscored by the finding that *S. meliloti* strains carrying extra copies of the riboflavin biosynthesis gene *ribBA* could release 15% more riboflavin than wild-type, and were 55% more efficient in alfalfa root colonization for nodule formation (Yang et al., 2002).

Physiologically, lumichrome has been shown to influence plant growth, but with differing effects depending on the plant species and metabolite concentration. Treating the roots of cowpea, Bambara groundnut, soybean, pea, lupin, sorghum, and maize plants with 10 nM purified lumichrome and 10 mL of infective rhizobial cells (0.2 OD_600_) for 44 h in growth chambers, increased stomatal conductance and leaf transpiration rates in cowpea, but decreased both parameters in Bambara groundnut, soybean, and maize, and had no effect on them in pea and sorghum (Matiru and Dakora, 2005b). In that study, the effect of bacterial inoculation closely mirrored that of 10 nM lumichrome application, again indicating that rhizobial effects on these physiological changes including stomatal functioning (whether in nature or under experimental conditions) were more likely due to the lumichrome molecule released by symbiotic rhizobia in the rhizosphere. Thus, the finding that rhizobial inoculation in the field alleviated the effects of water stress in symbiotic legumes (Figueiredo et al., 1999) could be attributed to strain secretion of lumichrome that decreased stomatal conductance and reduced plant water loss. More studies are needed to explore matching superior N_2_-fixing ability in inoculant rhizobia with high lumichrome production as insurance for increased water-use efficiency and drought tolerance in food legumes.

Developmentally, the supply of 5 nM lumichrome to roots of cowpea and soybean seedlings elicited early initiation of trifoliate leaf development, expansion in unifoliate and trifoliate leaves, and increased stem elongation, which together caused an increase in shoot and plant total biomass relative to the control (Matiru and Dakora, 2005a). Even with monocots such as maize and sorghum, lumichrome application at 5 nM also induced leaf area expansion, and thus increased shoot and total biomass, but had no effect on the leaf area of some cereals. Similar plant growth data were also obtained with lumichrome supply to lotus and tomato (Gouws et al., 2012). Other developmental changes observed included an increase in root growth in sorghum, millet, lotus and tomato caused by the supply of 5 nM lumichrome to seedlings of these species (Matiru and Dakora, 2005a; Gouws et al., 2012). Higher doses of lumichrome at 50 nM however depressed the development of unifoliate leaves in soybean, the second trifoliate leaf in cowpea, and shoot biomass in soybean. Furthermore, the 50 nM concentration also consistently decreased root development in cowpea and millet, but had no effect on the other species (Matiru and Dakora, 2005a). These findings also showed that the developmental effect of lumichrome on plant species was not age-specific as growth of both 11- and 37-day-old sorghum, 23- and 37-day-old soybean, 23- and 37-day-old millet, as well as 11- and 37-day-old cowpea were significantly increased by lumichrome supply at 5 nM concentrations. Unlike the legumes, however, the supply of 5 nM lumichrome markedly increased (*P* < 0.05) root growth in cereals such as sorghum and millet (Matiru and Dakora, 2005a). From these results, lumichrome is no doubt a rhizosphere signal molecule that affects seedling development in both monocots and dicots. It is likely that, in nature, lumichrome released by symbiotic rhizobia into the rhizosphere dictate the developmental path of plant species than is currently known, with potential for greater plant growth from increased water/mineral uptake and/or drought tolerance.

At the metabolic level, shoot and root application of lumichrome increased starch accumulation in roots of both lotus and tomato, which suggests a role for lumichrome in carbon partitioning and modulation of carbon fluxes in infected symbiotic plant cells (Gouws et al., 2012). This argument is re-inforced by the fact that lotus-treated roots showed a reduction in carbonaceous and nitrogenous solutes such as organic acids and amino acids. Root treatment with lumichrome also increased ethylene evolution rates in lotus, but not in tomato (Gouws et al., 2012). Taken together, these findings show that bacteria are capable of producing various simple organic molecules that serve as environmental cues in altering plant development. With the discovery of more active novel bacterial metabolites, it has become clear that besides the classical phytohormones such as auxins, cytokinins, gibberellins and abscisic acid, additional signal molecules exist that influence plant development. Although Phillips et al. (1999) attributed the enhanced plant growth from lumichrome application to increased net C assimilation via PEP carboxylase activity, the marked developmental changes (dramatic expansion in unifoliate and trifoliate leaves, and the increased stem elongation) observed with lumichrome application to cowpea and soybean would seem to suggest that this molecule stimulates plant growth via cell division and cell expansion, as happens with classical phytohormones (Mansfield, 1978; Ross et al., 2001; Campanoni et al., 2003; van der Graaff et al., 2003). In fact, it is our view that both lumichrome and riboflavin caused the developmental changes in plants by inducing the synthesis of classical phytohormones, which then modulate plant growth. However, experimental data are needed to support this claim.

## Agronomic Benefits of Lumichrome and Riboflavin

The increase in stem elongation, early initiation and rapid expansion of trifoliate leaves with the provision of 5 nM lumichrome to cowpea and soybean plants resulted in a twofold accumulation of dry matter in trifoliate leaves relative to 0-lumichrome control (Matiru and Dakora, 2005a). Lumichrome could also stimulate seedling development in monocots such as millet, sorghum and maize, in addition to legumes. As a result, whole-plant dry matter yield of these cereal species receiving 5 nM lumichrome was greater compared to control (Matiru and Dakora, 2005a). Root growth was also much greater in cereals (especially millet and sorghum) than legumes, suggesting that in the former, lumichrome application altered assimilate partitioning in favor of root development. Gouws et al. (2012) also observed an increase in dry matter accumulation following lumichrome application to lotus and tomato. The observed promotion in plant growth by lumichrome in both monocots and dicots suggests that, in addition to tapping symbiotic N contributed in cropping systems, cereals can also benefit from growth stimulation by lumichrome released by N_2_-fixing rhizobia in the soil. Its growth-promoting effect on both monocots and dicots further suggests that lumichrome is capable of influencing plant rhizospheres in both natural and agricultural ecosystems.

Foliar application of lumichrome at 10^–6^ M concentration significantly increased shoot and total dry matter yield of field-grown soybean plants (Khan et al., 2008). The increased accumulation of dry matter was partly due to a marked increase in leaf area with lumichrome supply (Khan et al., 2008). As found with cowpea and soybean, the observed increase in plant growth (Matiru and Dakora, 2005b) and Fe uptake (Matiru and Dakora, 2004) following sorghum inoculation with infective rhizobia could be attributed to lumichrome secreted by the introduced bacterial cells. As an agronomic practice, lumichrome supply with rhizobial inoculants therefore has the potential to increase crop yields in agricultural systems.

## Effect of N and P Nutrition on Rhizobial Exudation of Lumichrome, Riboflavin and IAA

There are a number of factors affecting the production and release of metabolites by soil bacteria. For example, the synthesis and extracellular release of lumichrome, riboflavin and IAA by rhizobia was found to differ between and among bacterial species and strains (Kanu et al., 2007). In some studies, there was generally greater production of lumichrome, riboflavin and IAA by N_2_-fixing bacteria than those unable to nodulate legumes such as *Psoralea pinnata* and sirato (Shokri and Emtiazi, 2010; Kanu and Dakora, 2012), a finding consistent with their role in symbiotic N_2_ fixation (Phillips et al., 1999; Lambrecht et al., 2000; Matiru and Dakora, 2005a,b; Gouws et al., 2012). In fact, Kanu and Dakora (2012) found that strain TUT57pp, which was effective in N_2_ fixation, produced 2.2-fold and 3.2-fold more IAA than the non-nodulating isolates TUT65prp and TUT33pap, respectively. Furthermore, studies on the effect of lumichrome on N and P nutrition in rhizobial isolates showed that N_2_-fixing strain TUT57pp consistently produced significantly more lumichrome, riboflavin and IAA than its non-nodulating counterpart TUT61pp (Kanu and Dakora, 2009, 2012). These results provide further evidence that the three molecules (lumichrome, riboflavin and IAA) are indeed rhizobial symbiotic signals.

Although we know the effect of N and P nutrition on Nod factor production in symbiotic rhizobia (McKay and Djordjevic, 1993), little information currently exists on the effects of these mineral nutrients on the biosynthesis of other symbiotically-important metabolites such as lumichrome, riboflavin and IAA. There are reports of marked variation in the secretion of lumichrome, riboflavin and IAA by symbiotic rhizobia compared to their non-nodulating bacterial counterparts (Shokri and Emtiazi, 2010; Kanu and Dakora, 2012). Kanu and Dakora (2012) measured much greater concentrations of lumichrome and riboflavin in the culture filtrate of five N_2_-fixing and 11 non-nodulating bacterial strains grown at high P (5.7 mM) than at low P (1.4 mM). The five N_2_-fixing isolates also differed in their levels of extracellular secretion of lumichrome, with TUT23prt releasing the most lumichrome at both low P and high P, and TUT18pac the least. Strain TUT23prt would therefore seem to be more adaptable to environments with a wide range of P concentrations, a trait very useful for selecting food legumes for P tolerance. The subtle differences in strain adaptation found between TUT23prt and TUT18pac point to why some legume/rhizobial symbioses perform well across environments with varying nutrient regimes, and hence in the case of P, the commonly encountered low-P tolerant and low-P sensitive symbioses.

Ammonium nutrition (whether at 28.1 mM or 112.0 mM NH_4_^+^) had no effect on the biosynthesis and release of riboflavin by rhizobia (Kanu and Dakora, 2012), a finding consistent with the reported lack of response of Nod factor secretion to ammonium supply (McKay and Djordjevic, 1993). However, lumichrome production was markedly affected by ammonium nutrition (Kanu and Dakora, 2012). While some strains produced more, or less, lumichrome with ammonium supply, strains TUT23prt and TUT33pap produced significantly large amounts of lumichrome at both low and high ammonium concentrations (Kanu and Dakora, 2012), a trait that could contribute to the strains’ tolerance of high soil N. The level of lumichrome and riboflavin production by the test isolates from *Psoralea* species also differed with nitrate nutrition. Feeding these strains with 59.3 mM nitrate resulted in significantly decreased concentration of lumichrome and riboflavin in bacterial exudates (Kanu and Dakora, 2012), indicating an inhibitory effect of nitrate on the biosynthesis and extracellular release of the two metabolites by rhizobial bacteria. In fact, the levels of lumichrome in culture filtrate were decreased by high nitrate concentration for all the isolates. A similar decrease in Nod factor production was observed by McKay and Djordjevic (1993), following nitrate supply to *Rhizobium leguminosarum* bv. *trifolii*. It was interesting to note that, in the study by Kanu and Dakora (2012), the isolates which showed the least production of riboflavin at high nitrate (e.g., TUT10pm and TUT13pac), were also among the least in lumichrome production at high nitrate. More importantly, however, the observed inhibition of lumichrome and riboflavin biosynthesis and release by nitrate is in addition to its known depressive effect on nodulation and N_2_ fixation in symbiotic legumes (Streeter and Wong, 1988; Ayisi et al., 2000). In nature, soil nitrate at high concentrations is therefore likely to inhibit nodulation in legumes via its repressive effect on the synthesis and secretion of lumichrome and riboflavin by rhizobia, given the fact that the former was found to increase nodulation in lotus plants (Gouws et al., 2012).

## Effect of Rhizobial Strain, Temperature, Salinity, and pH on Bacterial Secretion of Lumichrome and Riboflavin

Metabolic adaptation plays a major role in the survival of legumes and their microsymbionts in harsh environments such as the nutrient-poor, acidic, dry and water-deficient soils of the Cape fynbos in South Africa. Kanu and Dakora (2009) found that bacterial isolates from *Psoralea* nodules collected from the fynbos differed in their levels of secretion of lumichrome, riboflavin and IAA, as well as in their exudation response to pH, salinity and temperature. For example, while isolate AS2 from *Psoralea* nodules could produce greater amounts of lumichrome at both pH 5.1 and 8.1, strains RT1 and P1 secreted more lumichrome per cell at only pH 8.1. Strains AP1 and RP2 were also found to produce more riboflavin at pH 8.1 than pH 5.1, while strain RT1 produced greater amounts of riboflavin at pH 8.1 than pH 5.1. Taken together, the estimated levels of lumichrome and riboflavin secreted by *Psoralea* bacterial isolates ranged from 0.1 to 15 nM (Kanu and Dakora, 2012). These variations in the concentration of lumichrome and riboflavin released by *Psoralea* isolates is consistent with the findings of earlier studies which showed significantly greater production of riboflavin by *Bradyrhizobium japonicum* Tal 110, *S. meliloti* RAKI and *Sinorhizobium fredii* 6217 relative to eleven other standard laboratory strains (Kanu et al., 2007). In contrast, *Rhizobium leguminosarum* bv. *viceae* 30, *Bradyrhizobium* CB756, and *Sinorhizobium arboris* lma 14919 exhibited the lowest production of lumichrome in culture filtrate when compared to the other test strains (Kanu et al., 2007).

As a further evidence of metabolic adaptation, two *P. repens* strains (RP1 and RP2) isolated from a very saline environment close to the Atlantic Ocean secreted large amounts of lumichrome and riboflavin at both low and high salinity levels (Kanu and Dakora, 2009). Although the concentration of IAA produced by *Psoralea* isolates was greater at high acidity and high temperatures, lumichrome production was more elevated at lower (10°C) than higher (30°C) temperature (Kanu and Dakora, 2009). The greater production of lumichrome at 10°C than 30°C was not surprising as Nod factors produced by *Bradyrhizobium aspalati* (now *Burkholderia tuberum*) isolated from *Aspalathus canosa* in the Cape fynbos was also greater at 12°C than 28°C (Boone et al., 1999). This can be explained by the fact that legume nodulation in the Mediterranean Cape region of South Africa occurs during the winter rains when temperatures are low, around 10–15°C. Thus, the biosynthesis and release of symbiotic signals such as Nod factors by rhizobia and flavonoid *nod* gene-inducers by the Cape legumes are likely to be metabolically more adapted to the lower (10°C) than higher (30°C) rhizosphere temperatures. As found with the biosynthetic response of lumichrome to salinity in the salt-tolerant *P. repens* from the Western Cape, legumes and their microsymbionts are generally metabolically-adapted to the environmental factors of their niches.

The observed variation in the secretion of lumichrome, riboflavin and IAA by bacterial isolates from *Psoralea* root nodules exposed to different pH, salinity and temperature regimes, or fed different levels of N (nitrate and ammonium) and P, was due to alteration in the number of bacterial cells. For example, the number of rhizobia measured as colony forming units (CFU) ranged from 0.91 to 121.48 × 10^7^ cfu mL^–1^ at pH 5.1 and from 0.69 to 214.05 × 10^7^ cfu mL^–1^ at pH 8.1 (Kanu and Dakora, 2009, 2012). This suggests that the genes encoding these metabolites are regulated differently by the imposed environmental factors. Furthermore, our findings indicate that natural changes in pH, salinity and/or temperature in plant rhizospheres could potentially elevate the concentrations of lumichrome, riboflavin and IAA in soils, with consequences for ecosystem functioning as both lumichrome and riboflavin (being vitamins) act as growth factors and developmental signals in plants, microbes and humans.

## Riboflavin as a Defense Molecule in Plants

Besides riboflavin and lumichrome, bacteria and plants produce other vitamins such as thiamine, biotin, niacin and ascorbic acid for their growth and cellular functioning. Recent studies have however revealed a new role for these vitamins in plant–microbe interactions, one being protection against pathogens (Mehboob et al., 2009; Palacios et al., 2014). It has been shown, for example, that spraying riboflavin (0.1 up to 10 mM concentration) on tobacco or *Arabidopsis* leaves caused resistance to *Peronospora parasitica*, *Pseudomonas syringae* pv. *Tomato*, Tobacco mosaic virus (TMV), and *Alternaria alternata* (Dong and Beer, 2000). In that study, riboflavin was found to induce expression of pathogenesis-related genes, leading to systemic acquired resistance to pathogens without the involvement of salicylic acid (Dong and Beer, 2000; Zhang et al., 2009; Liu et al., 2011).

In addition to its requirement as a growth factor for microbes, plants and humans, thiamine (vitamin B1) has also been reported to function as a defense molecule in inducing systemic acquired resistance in the plant kingdom (Ahn et al., 2005). This discovery that vitamins protect plants from pathogens has led to the suggestion that most root-colonizing, non-pathogenic, biocontrol bacteria probably elicit systemic acquired resistance in plants that is independent of the salicylic acid signaling pathway (Van Wees et al., 1997). Clearly, vitamins (especially riboflavin and its degradation product lumichrome) produced by microbes in the rhizosphere are probably the major elicitors of plant defense against soil-borne pathogens in the real world.

For example, rhizosphere microbes such as the fungus *Ashbya gossypii*, which overproduces riboflavin (Lim et al., 2001) probably provides a blanket protection to plants from its copious production and release of this molecule into the rhizosphere. But given the commonly known role of isoflavonoid phytoalexins and phytoanticipins in plant defense (Dakora and Phillips, 1996), it is likely that the overall health of a legume is dependent on molecular cross-talk involving the total defense appertoire of isoflavones, anthocyanins, riboflavin, thiamine and other yet unknown molecules. Whatever the case, we now know that vitamins such as riboflavin produced by rhizobia and other microbes have multiple functions, which include serving as (i) growth factors for microbes, plants and humans, (ii) signals for stomatal functioning in land plants, and (iii) protectants/elicitors in plant defense. So far, however, no study has found a role for lumichrome in plant defense.

## Is Mineral Nutrition in Nodulated Legumes Controlled by Multiple Symbiotic Signals via Molecular Cross-Talk?

One major finding from rhizobial interaction with monocot and dicot plant species is the effect of lumichrome on stomatal functioning. Matiru and Dakora (2005b) showed that applying 10 nM purified lumichrome, 10 nM ABA, or 10 ml of infective rhizobial cells at 0.2 OD_600_ to cowpea and lupin increased stomatal conductance and transpiration rates, but decreased them in soybean, Bambara groundnut and maize, and showed no effect in pea and sorghum. The decrease in stomatal conductance and transpiration with lumichrome supply to maize, soybean and Bambara groundnut closely mirrors the reduced stomatal conductance and leaf transpiration rates caused by elevated CO_2_ in C3 plant species (40.5 and 3.6%, respectively, in soybean; see Madhu and Hatfield, 2014). In one study, the decrease in stomatal conductance and transpiration rates with elevated CO_2_ led to reduced mineral ^15^N uptake (Kanemoto et al., 2009), just as the reduced stomatal conductance, and hence lower transpirational pull in test legumes exposed to elevated CO_2_ also resulted in significantly decreased uptake of Mg, Fe, Cu, and B (Duval et al., 2012). In contrast, where there was an increase in stomatal conductance and leaf transpiration, mineral nutrient uptake was also increased in roots. For example, Tani and Barrington (2005) reported an increase in the uptake of Cu and Zn by wheat from high transpiration rates, following irrigation, while Novák and Vidovic (2003) also found a direct relationship between N, P, and K uptake and leaf transpiration rates. Taken together, those findings indicate that soil mineral acquisition by plant roots is directly linked to leaf transpiration rates, stomatal conductance, and the water status of the rhizosphere. The parallel drawn here between the effect of elevated CO_2_ and lumichrome on stomatal functioning is that a decrease in stomatal conductance from elevated CO_2_ causes reduced transpiration rates and decreased nutrient uptake, while an increase in stomatal conductance from lumichrome supply elevates the transpirational pull and promotes mineral uptake and transport in the xylem stream. These findings clearly suggest that the uptake of mineral nutrients and their accumulation in plants is controlled by stomatal functioning, and hence by the factors that modulate stomatal opening and closure.

Plant water and nutrient relations are thus intimately linked to stomatal functioning, such that leaves close their stomata when the roots sense soil water deficit via organic molecules. Stomatal closure in response to water stress (be it drought or waterlogging) is signaled by simple metabolites such as lumichrome, riboflavin and ABA, which are produced more abundantly by symbiotic rhizobia than other bacterial endophytes (Kanu and Dakora, 2009, 2012). Because its accumulation in leaves has been associated with stomatal closure during waterlogging or drought (Jackson and Hall, 1987), ABA is perceived as the major molecule regulating stomatal function, a role confirmed in several experiments using ABA-deficient mutants and their wild types (Jackson and Hall, 1987).

However, recent studies have identified new players in stomatal functioning of symbiotic legumes. For example, applying lumichrome and infective rhizobial cells to plant roots increased stomatal conductance and transpiration rates in cowpea and lupin, which was similar to the ABA control treatment, but decreased them in soybean, Bambara groundnut and maize, as also found with ABA (Matiru and Dakora, 2005b). Stomatal functioning in pea and sorghum was however not affected by lumichrome and rhizobial application, or by ABA (Matiru and Dakora, 2005b). These changes in stomatal functioning in response to lumichrome, ABA, and infective rhizobial cells were so similar in all test plant species that lumichrome and ABA appeared to play an identical role in stomatal functioning. It therefore seems that lumichrome and ABA can act separately or collectively to achieve the same desired outcome in stomatal functioning, be it aperture closure or opening.

The identical effects of lumichrome and ABA on stomatal functioning therefore suggest molecular cross-talk by the two compounds in controlling stomatal closure and opening. As shown in Figure 2B, the transmission of root-to-shoot signals such as lumichrome, riboflavin, ABA, and possibly Nod factors, can individually or collectively cause an increase in stomatal conductance and greater transpirational pull, leading to enhanced water absorption and increased mineral uptake. Except for Nod factors, the presence of the other signals (lumichrome, riboflavin and ABA) in the xylem stream en route to photosynthetic leaves has already been confirmed in legumes (Jackson and Hall, 1987; Phillips et al., 1999; Matiru and Dakora, 2005b), and their accumulation in leaves of cowpea and soybean also established (Matiru and Dakora, 2005b). So far, however, no study has shown the presence of rhizobial Nod factors in the xylem sap of symbiotic legumes.

A recent report has revealed increased mineral accumulation in high N_2_-fixing cowpea genotypes than their low-fixing counterparts (Belane et al., 2014). The concentration of P in leaves of high N_2_-fixers was two-fold greater than the low-fixers. This increase in mineral accumulation could be attributed to a range of factors, which include (i) rhizobial exudation of metabolites (e.g., siderophores, IAA, ABA, and organic acids), (ii) host–plant secretion of root exudates that solubilize unavailable minerals (Dakora and Phillips, 2002), and (iii) plant/rhizobial release of growth-promoting molecules (Dakora, 2003) that increase root hair production and nutrient absorption. However, the increase in stomatal aperture induced by lumichrome, riboflavin and ABA followed by the concomitant increase in transpiration rates, which promoted mineral uptake (Novák and Vidovic, 2003; Tani and Barrington, 2005) suggests a direct role of these metabolites in the accumulation of nutrient elements by high N_2_-fixing cowpea varieties.

As a working model, we propose that lumichrome, riboflavin, ABA and possibly Nod factors secreted by symbiotic rhizobia in the rhizosphere get taken up by plant roots and translocated to shoots (Carpenter, 1943; Jackson and Hall, 1987; Matiru and Dakora, 2005b) where they elicit stomatal opening via molecular cross-talk (Figure 2B) in a concentration-dependent manner. That way, water and mineral uptake is enhanced. However, because the N_2_-fixing efficacy of rhizobial strains is directly linked to the quality and quantity of the secreted symbiotic signals, their molecular effect on stomatal functioning is also linked to the strains’ symbiotic efficiency. In fact, we have shown elsewhere that N_2_-fixing efficacy of rhizobia is correlated to leaf stomatal conductance of the host plant, and hence mineral accumulation in the legume. This relationship between strain symbiotic efficiency and stomatal functioning of the host plant is believed to control the symbiosis-induced accumulation of mineral nutrients in nodulated legumes (Belane et al., 2014).

A recent study has shown increased accumulation of ABA and IAA in lotus plants treated to lumichrome (Gouws et al., 2012). While such an increase in the formation of phytohormones in lumichrome-fed plants could help to explain the developmental changes associated with lumichrome application to plant roots, it could however also imply that these molecules cross-talk in their regulation of stomatal functioning, which leads to increased water and mineral uptake by roots. It is therefore our view that the symbiosis-induced accumulation of mineral nutrients in legumes (Belane et al., 2014) is due to the rhizosphere effect of lumichrome, riboflavin, IAA, ABA, and possibly Nod factors secreted by rhizobial bacteria (Figure 2B). The stomata in plants consist of specialized guard cells that regulate photosynthetic CO_2_ uptake and leaf transpiration (Chen et al., 2012; Hills et al., 2012; Liu et al., 2014). The guard cell slow anion channel (SLAC) gene is apparently the “master switch” for stomatal closure (Maierhofer et al., 2014; Zheng et al., 2014). But how lumichrome, riboflavin and ABA work together to induce stomatal opening or closure, and hence increase or decrease mineral uptake, is still unclear. However, the greater root proliferation caused by the application of lumichrome (5 nM) to sorghum and millet (Matiru and Dakora, 2005a), or to lotus and tomato (Gouws et al., 2012), can also increase nutrient uptake in plant species. Rhizobia and other rhizosphere diazotrophs probably play a much greater role in the mineral nutrition of legumes and non-legumes than previously imagined.

## Rhizosphere Ecology of Lumichrome, Riboflavin, and IAA Secreted by Rhizobia

In both natural and agricultural ecosystems, low or high production of lumichrome, riboflavin and IAA can have ecological consequences in ecosystem functioning. For example, an increase in root respiration induced by lumichrome and riboflavin from root-colonizing rhizobia can lead to an elevated concentration of rhizosphere CO_2_, which is needed for growth of rhizobial populations in soil (Lowe and Evans, 1962). Furthermore, the increase in rhizosphere CO_2_ concentration from lumichrome and riboflavin can also stimulate growth of vesicular-arbuscular fungi (Bécard and Piché, 1989; Bécard et al., 1992) and therefore promote the incidence of mycorrhizal symbiosis. These indirect benefits of lumichrome and riboflavin to legume symbioses via their effects on the plant are essential for enhancing N and P nutrition.

Furthermore, rhizobia and nodule endophytes isolated from eight *Psoralea* species (namely, *Psoralea pinnata*, *P. aphylla*, *P. aculeata*, *P. monophylla*, *P. repens*, *P. laxa*, *P. asarina*, and *P. restioides*) growing naturally in different locations within the Cape fynbos of South Africa exhibited large variations in their exudation of lumichrome, riboflavin and IAA (Kanu and Dakora, 2009, 2012), possibly due to bacterial adaptation to the localities where they were sampled. For example, two *P. repens* strains isolated close to the Atlantic Ocean secreted large amounts of lumichrome and riboflavin at both low and high salinity (Kanu and Dakora, 2009). Similarly, *Psoralea* isolates adapted to the acidic soils of the Cape fynbos also produced greater amounts of IAA even under very low pH conditions (Kanu and Dakora, 2009). The ability of native rhizobia to secrete symbiotic signals such as lumichrome, riboflavin and IAA under harsh environmental conditions implies that, even with climate change, indigenous legumes and their associated microsymbionts are unlikely to be affected in their symbiotic functioning.

Additionally, while most root-colonizing bacteria produce and release lumichrome and riboflavin (Phillips et al., 1999), others can synthesize and release eight times more extracellular riboflavin relative to their internal cellular concentration (Yang et al., 2002). These findings suggest that the two molecules have evolved directly or indirectly as rhizosphere signals influencing the outcomes of plant–bacterial interactions. It is clear from these studies that natural changes in pH, salinity and temperature within plant rhizospheres can elevate the concentrations of lumichrome, riboflavin and IAA in soils, with consequences for ecosystem functioning. For example, the high lumichrome production at 10°C than 30°C temperature (Kanu and Dakora, 2009) can alter nodulation and N_2_ fixation of legumes in the Mediterranean fynbos habitat, where winter rainfall supports plant growth, nodulation and N_2_ fixation.

## Conclusion

Bacterial exudation of the rhizosphere signals lumichrome, riboflavin and IAA can vary with rhizobial strain, salinity, soil temperature and pH. Lumichrome taken up by plant roots and transported to the shoots probably elicits the formation of morphogenic molecules that cause cell division, cell expansion and cell extensibility, leading to an increase in leaf expansion, and stem elongation. Rhizobial inoculation as well as lumichrome and ABA supply to plant roots induced identical effects on stomatal functioning in both monocots and dicots. The three treatments consistently increased, or decreased, stomatal conductance and transpiration rates depending on the plant species. Plant roots therefore seem capable of collecting environmental signals from soil in the form of simple organic molecules released by microbes, and using them to adapt to their niches. An increase in the concentration of ABA and IAA in organs of lotus plants supplied with lumichrome (Gouws et al., 2012) could suggest that the observed developmental changes caused by lumichrome application to roots of monocots and dicots (Matiru and Dakora, 2005a) was probably due to increased levels of phytohormones elicited by the applied lumichrome. This however remains speculative in the absence of any genetic studies on the molecular basis for plant responses to lumichrome and riboflavin. Future studies need to address many unanswered questions. For example, what are the mechanisms underlying plant growth stimulation by lumichrome and riboflavin? Will rhizobial inoculation elicit same response in both legume and non-legume species as observed with lumichrome application to roots of monocots and dicots? Future experiments should quantify classical phytohormones such as ABA, IAA, cytokinins and gibberellins in lumichrome and riboflavin-treated plants in order to unravel the mechanisms underlying plant response to these bacterial metabolites, and in so doing, add to our current understanding of the functioning of bacterial metabolites in plant rhizospheres. The relationship between rhizobial inoculation, leaf stomatal functioning, and mineral accumulation also need to be further explored.

### Conflict of Interest Statement

The authors declare that the research was conducted in the absence of any commercial or financial relationships that could be construed as a potential conflict of interest.
